# Idiopathic normal pressure hydrocephalus: associations between CSF biomarkers, clinical symptoms, and outcome after shunt surgery

**DOI:** 10.1186/s12987-025-00661-w

**Published:** 2025-05-19

**Authors:** Majd Saadaldeen, Anna Jeppsson, Per Hellström, Kaj Blennow, Henrik Zetterberg, Carsten Wikkelsø, Mats Tullberg

**Affiliations:** 1 Hydrocephalus research unit, Department of Clinical Neuroscience, Institute of Neuroscience and Physiology, Sahlgrenska Academy, University of Gothenburg, Gothenburg, Sweden; 2 Department of Neurology, Sahlgrenska University Hospital, Gothenburg, Sweden; 3 Clinical Neurochemistry Laboratory, Sahlgrenska University Hospital, Mölndal, Sweden; 4 Department of Psychiatry and Neurochemistry, Institute of Neuroscience and Physiology, Sahlgrenska Academy, University of Gothenburg, Gothenburg, Sweden; 5 Department of Neurodegenerative Disease, UCL Institute of Neurology, Queen Square, London, UK; 6 UK Dementia Research Institute at UCL, London, UK; 7 Hong Kong Center for Neurodegenerative Diseases, Clear Water Bay, Hong Kong, China; 8 Wisconsin Alzheimer’s Disease Research Center, School of Medicine and Public Health, University of Wisconsin, University of Wisconsin-Madison, Madison, WI USA; 9 Pitié-Salpêtrière Hospital, Paris Brain Institute, ICM, Sorbonne University, Paris, France; 10 Neurodegenerative Disorder Research Center, Division of Life Sciences and Medicine, Department of Neurology, Institute on Aging and Brain Disorders, University of Science and Technology of China and First Affiliated Hospital of USTC, Hefei, P.R. China

**Keywords:** Idiopathic normal pressure hydrocephalus, Biomarkers, CSF, Cerebrospinal fluid, Symptoms, Outcome

## Abstract

**Background:**

The neurochemical alterations in cerebrospinal fluid (CSF) associated with the typical symptomatology in idiopathic normal pressure hydrocephalus (iNPH) and their association with outcome after shunt surgery are unsettled.

**Aim:**

To explore associations between concentrations of CSF biomarkers reflecting amyloid- and tau pathology, neuronal degeneration as well as astrocytic activation and the characteristic symptomatology in iNPH and to examine whether these biomarkers can predict the postoperative outcome in all patients and in patients without evidence of Alzheimer’s disease (AD) pathology.

**Methods:**

This explorative study included 81 patients diagnosed with iNPH at the Hydrocephalus research unit, Sahlgrenska. Symptoms were assessed using the iNPH-scale and standardized clinical tests measuring gait, balance, cognition and urinary incontinence before and median 8 months after shunt surgery. Pre-operative lumbar CSF concentrations of Aβ38, Aβ40, Aβ42, ratio Aβ42/Aβ40, sAPPα, sAPPβ, T-tau, P-tau, MCP-1, and NFL were analyzed. A low Aβ42/Aβ40 ratio defined patients with AD pathology. Correlation and regression analyses between biomarker concentrations and clinical symptoms at baseline as well as postoperative change in symptoms after surgery, were performed.

**Results:**

Higher NFL correlated with more pronounced impairment in all clinical tests, i.e. included measures of gait, balance, cognition and urinary incontinence (r_p_=0.25–0.46, *p* < 0.05). Higher T-tau and P-tau correlated with poorer performance in cognitive tests (r_p_=0.26–0.39, *p* < 0.05). No biomarker could differentiate between improved and unimproved patients in the whole sample or in AD-pathology negative patients. Low ratio Aβ42/Aβ40 lacked predictive value. A higher preoperative P-tau was weakly correlated with less pronounced overall clinical improvement (r_p_ = -0.238, *p* = 0.036).

**Conclusions:**

Axonal degeneration, as indicated by elevated NFL, is probably involved in the generation of the full iNPH tetrade of symptoms and tau pathology more specifically with iNPH cognitive impairment. No CSF biomarker could identify shunt responders. CSF evidence of Alzheimer pathology should not be used to exclude patients from shunt surgery.

## Background

Idiopathic normal pressure hydrocephalus (iNPH) is a chronic, communicating form of hydrocephalus [1]. This disorder affects the elderly population with an estimated prevalence of 3% among individuals over 65 years and 5,9% among individuals over 80 years old [2, 3] and a prevalence of 1.5% was recently reported in 70-year olds [3]. The symptomatology includes gait and balance disturbance, cognitive impairment and urinary incontinence [1, 4]. Patients benefit from shunt surgery with clinical improvement seen in up to 80% of cases [5–7].

Identifying patients with iNPH eligible for surgery remains a challenge since predictive tests that have both high positive and negative predictive values are lacking [8], which together with a lack of specific diagnostic tests, leads to both underdiagnosis and undertreatment [9]. Diagnosis is based on identification of typical symptomatology and imaging findings. The iNPH-scale introduced by Hellström et al. [10] is a clinical scale developed to assess the specific symptomatology and postoperative outcome in iNPH.

The pathophysiology of iNPH is still not fully understood. A CSF dynamic disturbance is a prerequisite and vascular factors such as hypertension, diabetes, hyperlipidemia [11] and ischemia in the white matter of the brain [12, 13] as well as genetic mechanisms [14] are probably involved in the development of disease. Comorbidity is common in patients with iNPH and it has been suggested that coexisting neurodegenerative disease such as Alzheimer’s disease (AD) or cerebrovascular disease may explain why some patients do not improve after surgery [15–17].

CSF biomarker concentrations reveal neurochemical alterations related to pathophysiology in neurological and neurodegenerative disorders and are widely used for diagnostic and predictive purposes. We reported amyloid mis-metabolism in iNPH patients with a downregulation of amyloid cascade proteins but no or only minor neuronal degeneration and that a specific biomarker pattern of low T-tau and APP-derived proteins in combination with high Monocyte chemoattractant protein 1 (MCP-1) could distinguish iNPH from healthy individuals and clinical mimics [18]. Studies exploring associations between CSF biomarkers and clinical symptoms in iNPH are scarce. Lukkarinen et al. found that baseline CSF T-tau, P-tau and NFL correlated negatively with MMSE scores and that NFL correlated negatively with gait velocity, indicating that tau pathology and axonal degeneration may be involved in the generation of symptoms [19]. The predictive value of CSF biomarkers has yet to be established. Kazui et al. found that increased T-tau/Aβ42 as a measure of comorbid AD pathology was related to poorer shunt response in iNPH patients, whereas other studies reported no negative influence of AD pathology on outcome [20]. In the study by Lukkarinen et al., T-tau, P-tau and NFL also correlated negatively with outcome in MMSE. Braun et al. recently reported that higher levels of NFL and T-tau were weakly associated with less improvement after shunt surgery [21].

The aim of this study was to explore associations between CSF biomarkers describing a range of pathophysiological mechanisms including amyloid pathology (Aβ38, Aβ40, Aβ42, ratio Aβ42/Aβ40, sAPPα, sAPPβ), tau pathology (T-tau, P-tau), astrocytic activation (MCP-1) as well as axonal degeneration (NFL) and the core symptomatology of iNPH accessed by the iNPH scale as well as a number of single clinical measures, and to evaluate whether these biomarkers could predict the clinical outcome after shunt surgery in iNPH patients and also in the AD pathology negative patients.

## Methods

### Sample and data collection

This is a single center study from the Hydrocephalus Unit, Sahlgrenska University Hospital including 81 patients consecutively diagnosed with iNPH according to international guidelines [22] during 2007–2012 and subjected to shunt surgery. Data were collected retrospectively and all patients who were both pre- and postoperatively examined were included, given that sufficient CSF samples were available for analysis. Patients with missing postoperative data due to no-show at the follow up examination were excluded.

The patients underwent a standardized clinical examination with tests performed by a neurologist, a physical therapist, a neuropsychologist, and an MRI of the brain at baseline and approximately 6 months postoperatively. Evans’ index > 0.3 was mandatory for diagnosis. Decisions for surgery were made at multidisciplinary conferences. In patients for whom the surgical outcome was considered doubtful, mostly due to unclear or atypical symptomatology, the CSF tap test, or a lumbar infusion test was used as supplementary tests to strengthen the indication for shunt treatment and in such cases, only patients showing a positive tap test, or an increased R_out_ were selected for shunt surgery.

All patients received a ventriculo-peritoneal shunt with a Medtronic PS Medical Strata adjustable valve set at 1.5. All shunts were checked for patency at the postoperative follow-up based on clinical improvement and radiological findings. If doubts regarding the shunt remained following CT or MRI, mainly due to lack of clear clinical response, a radionuclide shuntography [23] or a lumbar infusion test was performed to ensure function.

### Clinical measures included in the study

The overall and specific symptom domain burden was measured using the iNPH-scale introduced by Hellström et al., yielding a total score and subscores for the four symptom domains of gait, balance, continence and cognition. The scale ranges from 0 to 100, where 100 equals the median expected performance of a healthy elderly person aged 70–74 years, while 0 means maximal burden of disease. Patients showing a postoperative increase of at least 5 iNPH scale points were classified as improved [10].

Gait function was also measured on the ordinal scale included in the iNPH scale as 1–6: 1 = Normal gait, 2 = Unsteady without using walking aids, 3 = Walking with a cane, 4 = Walking with a roller, 5 = Walking only if supported by other person/-s, or 6 = Wheelchair bound [10]. To assess cognitive function in more detail, specific tests for cognition were also analyzed, including Identical forms test (perceptual speed and accuracy) and Bingley’s visual memory test [24] as well as the four tests included in the iNPH scale cognitive domain: Grooved peg board, the Swedish Stroop test (Stroop colour and Stroop interference) and the Rey Auditory Verbal Learning test (RAVLT) [10]. For evaluation of impaired wakefulness and increased need of sleep, which has earlier been reported in iNPH patients [25, 26], average daily need of sleep in hours and evaluation of the organic mental disorder Somnolence Sopor Coma Disorder (SSCD) according to the system for classification of organic psychiatric disorders introduced by Lindqvist and Malmgren [27] scored on a 4 level ordinal scale (1 = absent, 2 = mild, 3 = moderate, 4 = severe) were recorded in line with our earlier studies [25, 26, 28].

### Analysis of CSF biomarker concentrations

A lumbar puncture was performed with the patient in the lateral recumbent position according to standard protocol to collect CSF samples, which were aliquoted and then kept frozen at -80 °C until analyzed. All the CSF samples were analyzed for research purposes batchwise in one round of experiments by board certified laboratory technicians who were blinded to the clinical data at the Clinical Neurochemistry Laboratory, Sahlgrenska University Hospital. The Amyloid precursor protein derivates Aβ38, Aβ40, Aβ42 and soluble APPβ, soluble APPα as well as MCP1 were all analyzed using electrochemiluminescence assays as described by the kit manufacturer (Meso Scale Discovery, Rockville, MD, USA) [29]. CSF T-tau and P-tau 181 concentrations were measured using commercially available INNOTEST ELISAs as described by the kit manufacturer (Fujirebio, Ghent, Belgium). Neurofilament light was measured using an in-house ELISA as previously described in detail [30].

### Statistical methods

Descriptive statistics were used for distributions of data. Scatter plots were made to visualize possible relationships between each CSF biomarker and the total score on the iNPH-scale preoperatively. Since the distributions of T-tau, P-tau and NFL were highly skewed, these data were logarithmized (ln) before entered in correlation and regression analyses. Pearson correlation coefficients (r_p_) were calculated to detect associations between the biomarkers as quantitative predictors, and the total score on the iNPH-scale as a quantitative outcome. Univariate regression analysis was performed with the total iNPH scale score at baseline or postoperative change in total iNPH scale score as dependent variable, and for calculating the estimated effect of each biomarker as well as a 95% confidence interval. Variables were controlled for normal distribution. To select significant biomarkers for the multivariable regression, the α-value was set to 0.1 to minimize the risk of type II error. Multivariable regression analysis was performed for the biomarkers with adjustment for possible confounders. An independent sample Mann-Whitney U-test was used to analyze the differences in data across groups such as differences in biomarker concentrations between the improved and non-improved sample groups. Moreover, correlation and univariate regression analyses were performed as beforementioned to examine possible associations between single biomarkers, as well as combinations of biomarkers, and the total outcome score as defined by the difference in the iNPH-scale before and after surgery. The patients were divided into subgroups of AD-positive or -negative pathology as defined by the CSF A*β*42/A*β*40-ratio with cut-off point set to 0.09 based on eye-ball test appreciation of the bimodal distribution and in alignment with previous findings [31, 32], i.e. patients with a high ratio > 0.09 were considered AD-negative. Fisher’s exact test was used to compare frequences of categorical data across groups of AD-negative and -positive patients. All tests were two-tailed and a p-value < 0.05 was considered significant. All statistical analyses were performed using IBM SPSS Statistics for Mac version 29.

## Results

Patients’ mean age was 73.3 years, 64% were males and 59% were improved by at least 5 points on the iNPH scale. Fifty patients were AD-negative, and this group showed similar baseline characteristics and responder rate as the 31 AD-positive patients (Table 1).

**Table 1:** Demographic data of the 81 patients with idiopathic normal pressure hydrocephalus

	All (*n* = 81)	AD negative (*n* = 50)	AD positive (*n* = 31)	
Age, mean (range)	73.3 (52–89)	72.3 (7.3)	74.8 (56–86)	*p* = 0.10
Sex, n (%)				
Female Male	29 (36%) 52 (64%)	15 (30%) 35 (70%)	14 (45%) 17 (55%)	*p* = 0.23
Risk factors, n (%)				
Hypertension Cardiovascular disease Diabetes	43 (53%) 26 (33%) 18 (23%)	29 (58%) 18 (36%) 14 (28%)	14 (47%) 8 (27%) 4 (13%)	*p* = 0.36 *p* = 0.46 *p* = 0.17
MMSE, mean (SD) Preop iNPH scale score, mean (SD)	23.6 (4.4) 47.8 (20.3)	24.2 (4.0) 49.7 (21.3)	22.7 (4.9) 45.1 (18.9)	*p* = 0.21 *p* = 0.49
Improved, n (%)	48 (59%)	40 (60%)	18 (58%)	*p* = 1.0

MMSE = Mini Mental State Examination. AD = Alzheimer’s disease pathology defined by CSF Aβ42/Aβ40-ratio. AD negative and AD positive patients are defined by Aβ42/Aβ40-ratio above or below 0.09 respectively. Improved = postoperative iNPH scale score increase of at least 5 points. P-values are given for comparisons of AD negative vs. AD positive patients.

Data for the clinical measures, available in 67–81 of the patients, are presented in Table 2. Biomarker concentrations were available in all 81 patients, except for T-tau (*n* = 76) and P-tau (*n* = 78).

**Table 2:** Pre- and postoperative iNPH scale scores and preoperative scores of of 81 patients with iNPH by symptom domain and total

INPH scale symptom domain	Pre-op score (Mean)	Post-op score (Mean)	Change score (Mean)	*P*-value
**Gait**	38.6	57.2	18.9	< 0.001
**Balance**	60.4	65.9	5.1	< 0.001
**Cognition**	47.3	57.7	8.7	< 0.001
**Urgency**	57.7	66.5	9.9	< 0.001
**Total iNPH-scale score**	47.9	60.3	12.4	< 0.001
**Other pre-op measures**				
**Bingley (0–12)**	4.1			
**Identical forms test (0–60)**	11.7			
**Daily need of sleep (h)**	9.3			
**SSCD-scores (1/2/3/4**,** frequency)**	30/16/7/3			

SD = Standard deviation. Maximum points on the iNPH-scale = 100 representing normal performance. Since the formula can be adjusted for the number of available domains, the total score was available for all patients despite missing data in individual symptom domains. D represents outcome scores total and for each domain, i.e. postop. score– preop. score. h = hours; SSCD = somnolence sopor coma disorder.

### Associations between biomarkers and the overall symptom burden at baseline

**Fig. 1:**
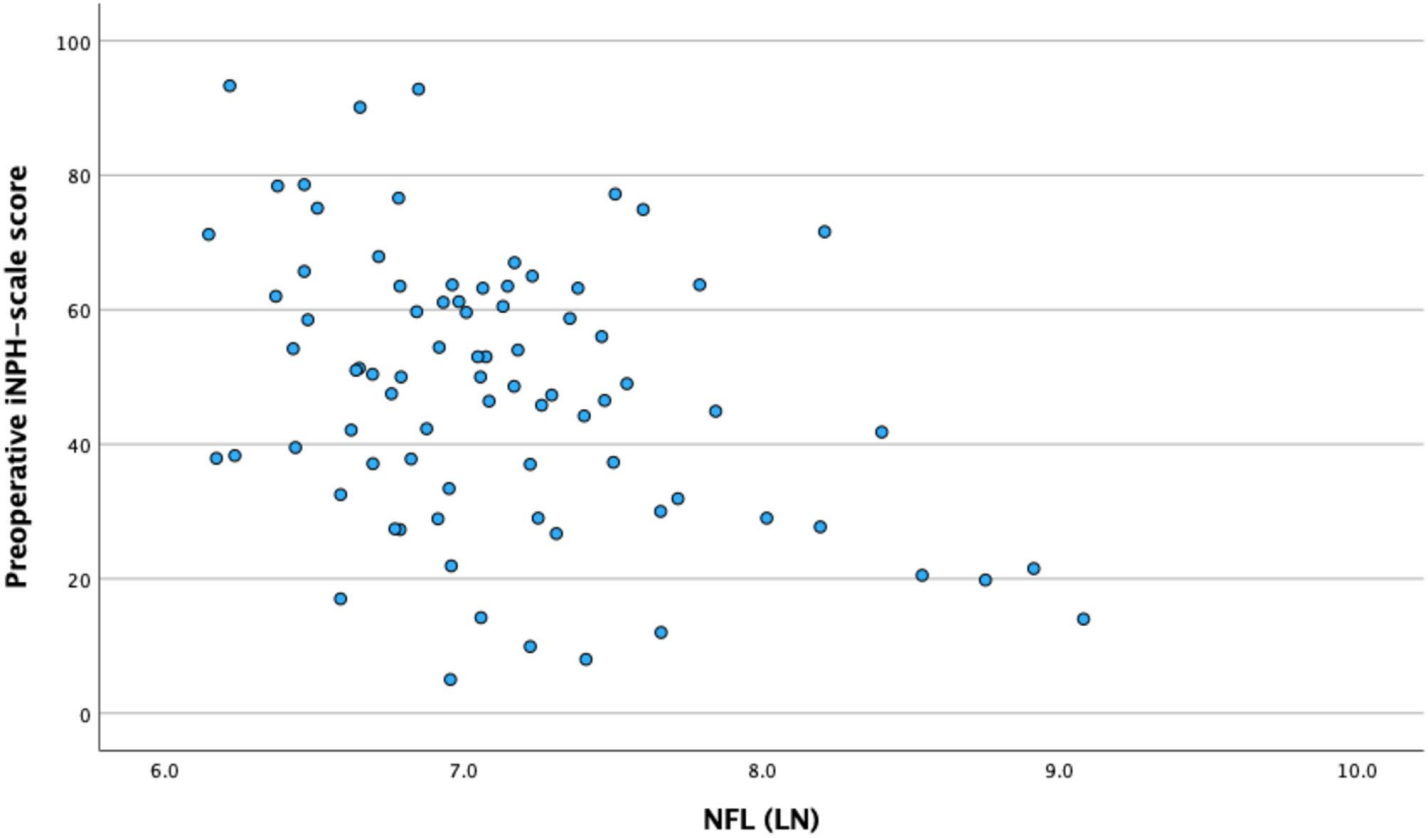
Scatter plot for NFL and the total iNPH scale score before surgery. R_p_= -0.375. Nota bene: logarithmic values for NFL

There was an inverse correlation between T-tau (r_p_= -0.30, *p* = 0.007), NFL (r_p_= -0.38, *p* < 0.001) and Aβ40 (r_p_= -0.24) and the total iNPH-scale score before surgery (Table 3; Fig. 1). However, none of the biomarkers presented a strong explanation of the variance in the total iNPH-score as the r^2^ never exceeded 0.15, the strongest predictor being NFL (explaining 13% of the variance). The r^2^ and 95% confidence intervals for each of the biomarkers are presented in Table 3.

Age was significantly correlated with Aβ38 (r_p_=0.303, *p* = 0.006), Aβ40 (r_p_=0.382, *p* < 0.001), Aβ42 (r_p_=0.296, *p* = 0.007), T-tau (r_p_=0.306, *p* = 0.007), P-tau (r_p_=0.232, *p* = 0.040), and NFL (r_p_=0.479, *p* < 0.001). Sex (*p* = 0.269) was not a significant confounder, and hence not adjusted for. In the multivariable regression analysis adjusted for age, there were no significant predictors. P-values for the three models are displayed in Table 3.

**Table 3:** Regression analyses with the biomarkers as independent variables and the total iNPH-scale score before shunt surgery as dependent variable

Biomarker	Standardized B	95%CI for B	Adjusted *r*^2^	*p*-value	*p*-value	*p*-value
		(Lower– upper)		**Univariable regression**	Multivariable regression*	**Adjustment for age**
Aβ38	-0.210	-0.017–0.000	0.032	0.060	0.243	0.567
Aβ40	-0.241	-0.008–0.000	0.046	**0.030**	0.373	0.710
Aβ42	-0.204	-0.063–0.002	0.029	0.068	0.418	0.603
sAPPα	0.110	-0.013–0.039	0.000	0.330	-	
sAPPβ	0.128	-0.016–0.061	0.004	0.255	-	
MCP-1	-0.096	-0.060–0.024	-0.003	0.395	-	
ln-T-tau	-0.304	-22.47– -3.586	0.080	**0.007**	**0.047**	0.110
ln-P-tau	-0.195	-24.24–1.662	0.025	0.087	0.138	0.192
ln-NFL	-0.375	-19.317– -5.563	0.130	**< 0.001**	0.351	0.565

*Biomarkers with p-values < 0.1 were included in the multivariable regression. Significant p-values in bold. Adjustment for age = multivariable regression with adjustment for age.

### Associations between CSF biomarkers and individual symptoms

A higher NFL correlated with poorer performance in all iNPH scale domains and all clinical tests at a weak-moderate strength (r_p_=0.25–0.51), with the strongest correlations for cognitive tests. A higher T-tau and P-tau correlated with poorer performance on the cognitive domain and on individual cognitive tests (r_p_=0.30–0.39) (Table 4).

**Table 4:** Correlations stronger than r_p_=0.25 between preoperative symptom scores and biomarker concentrations

	NFL(ln)	T-tau(ln)	*P*-tau(ln)	Aβ42
**Cognition (iNPH-scale)**	-0.36**	-0.39**	-0.26*	
Identical forms test (0–60)	-0.25*	-0.39***	-0.33**	
Bingley (0–12)	-0.29**	-0.34**		
SSCD (1–4)	0.46***	0.35**	0.31**	
Daily need of sleep (h)	0.35**	0.30*		
Stroop C Stroop I	0.34** 0.51**	0.37**	0.26*	
Grooved pegboard	0.38**	0.31*		
RAVLT	-0.33**	-0.39**	-0.30**	
**Gait (iNPH scale)**	-0.27*			-0.25*
Ordinal gait rating (1–6)	0.28*			
**Balance (iNPH scale)**	-0.39**			
**Urgency (iNPH scale)**	-0.25*			

Nota bene: logarithmic values for NFL, T-tau and P-tau. *=*p* < 0.05; **=*p* < 0.01; ***=*p* < 0.001. iNPH scale domain scores in bold. All correlations represent associations where a higher biomarker concentration is associated with a poorer test performance.

### Associations between baseline CSF biomarker concentrations and postoperative outcome

There were no significant differences in biomarker concentrations across groups of improved and non-improved (Table 5). A higher P-tau correlated weakly with less overall postoperative improvement (r_p_ = -0.238, *p* = 0.036). Age or sex was not associated with outcome. Since the univariable regression showed only one CSF biomarker association with *p* < 0.1, a multivariable regression was not performed. (Table 6).

**Table 5:** CSF biomarkers across subgroups of improved and non-improved patients

Biomarker (pg/mL)	Total (Mean)	Improved (Mean)	Unimproved (Mean)	*p*-value
**Aβ38**	1514.9	1549.98	1463.95	0.722
**Aβ40**	3780.4	3847.31	3683.02	0.758
**Aβ42**	361.6	368.56	351.52	0.551
**sAPPα**	441.3	469.21	400.64	0.134
**sAPPβ**	317.3	335.29	291.06	0.162
**MCP-1**	489.9	490.29	489.30	0.916
**T-tau**	237.5	236.76	238.74	0.410
**P-tau**	31.3	30.64	32.26	0.516
**NFL**	1571.0	1509.93	1659.88	0.263

**Table 6:** Univariable regression analysis with CSF biomarkers as independent variables and the postoperative change in total iNPH scale score as dependent variable

Biomarker	Standardized B	95% CI for B	Adjusted *r*^2^	*p*-value
		(Lower– upper)		**Univariable regression**
Aβ38	-0.004	-0.007– 0.007	-0.013	0.969
Aβ40	-0.034	-0.004–0.003	-0.011	0.761
Aβ42	0.036	-0.023–0.032	-0.011	0.752
sAPPα	-0.007	-0.022–0.021	-0.013	0.762
sAPPβ	0.013	-0.030–0.034	-0.012	0.912
MCP-1	-0.032	-0.040–0.030	-0.012	0.780
ln-T-tau	-0.165	-14.72–2.370	0.014	0.154
ln-P-tau	-0.238	-22.73– -0.817	0.044	**0.036**
ln-NFL	-0.106	-8.981–3.183	-0.001	0.346

**Fig. 2:**
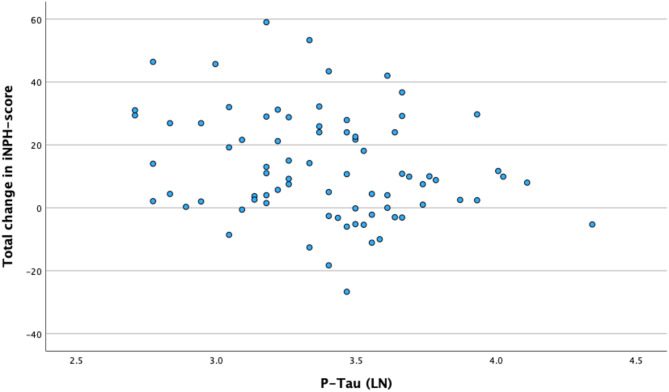
Scatter plot for P-tau and the overall outcome score defined as postoperative change in total iNPH scale score. r_p_=-0.238. Nota bene: logarithmic values for P-tau

A scatter plot and exact regression equation for P-tau and the total outcome score is presented in Fig. 2. Although linear negative tendency, the slope is not strongly inclined, yielding a weak correlation coefficient (r_p_) of only − 0.238.

### Associations between baseline CSF biomarker concentrations and postoperative outcome in AD-pathology negative iNPH patients

To rule out possible effects of AD-related pathology, the same analyses were made including only AD-pathology negative (A*β*42/A*β*40 ratio > 0.09) patients (*n* = 50): no significant associations were found between CSF biomarker concentrations, including P-tau-concentration, and the outcome after shunt surgery (Table 7).

**Table 7:** Regression analyses in AD-negative patients (*n* = 50) with the biomarkers as independent variables and the total outcome score after shunt surgery as dependent variable showing the strongest associations

Biomarker	Standardized B	95% CI for B (lower-upper)	Adjusted *r*^2^	*p*-value Univariable regression
ln-T-Tau	-0.159	-19.50–6.10	0.003	0.297
ln-P-tau	-0.270	-30.80–1.06	0.052	0.067
ln-NFL	-0.071	-8.73–5.30	-0.016	0.625

ln = logarithmized values

## Discussion

We explored possible associations between pre-operative lumbar CSF concentrations of biomarkers reflecting amyloid and tau pathology, neuronal degeneration, astrogliosis as well as AD-pathology and the baseline iNPH symptomatology and postoperative outcome in a sample of iNPH patients. No biomarker showed a strong association with baseline clinical presentation or overall improvement after shunt surgery or could distinguish between improved and unimproved patients. A higher NFL correlated with poorer performance in all assessed iNPH symptom measures, whereas a higher T-tau and P-tau correlated with poorer cognitive test performance. A higher P-tau was weakly correlated to less pronounced improvement. AD-pathology negative patients showed similar results.

### Associations between CSF biomarkers and clinical symptoms at baseline

Higher concentrations of NFL, T-tau, and Aβ40 correlated with a more pronounced overall symptom burden, defined as total iNPH scale score, NFL showing the strongest correlation. In individual symptom domains and clinical measures, NFL correlated with all test results, whereas T-tau correlated with the cognitive tests. All correlations were however weak. This suggests involvement to some degree of axonal degeneration, tau pathology and altered amyloid metabolism in the generation of symptoms in iNPH which corroborates previous studies [19, 33], axonal degeneration or dysfunction probably being the more paramount pathophysiological features. Kudo et al. reported a significant correlation between CSF T-tau and the severity of dementia and urinary incontinence in a study including secondary and idiopathic NPH patients and narrower clinical scales ranging between 0 and 2 [34]. Similarly, higher levels of NFL have previously been found to be associated with more symptoms in patients with NPH (i.e., both secondary and idiopathic cases) [33]. Further, a higher P-tau correlated with poorer performance in several cognitive tests, supporting the notion that tau pathology, in addition to axonal degeneration, is associated with cognitive decline.

We found age to be correlated with all CSF biomarkers except sAPPα, sAPPβ and MCP-1 (data not shown) which corroborates previous reports [35]. In multivariable analyses adjusted for age, none of the associations with overall symptom burden remained. Mattsson et al., in a larger study, investigated the role of age in AD-related CSF biomarkers, i.e. Aβ42, T-tau and P-tau and concluded that there was an age-dependent effect on the levels of these biomarkers, complicating the differentiation between elderly patients with AD and the control group as compared to the younger patient/control group, since the normal ageing process and AD-related pathology presented similar changes [36]. Stratifying our study sample by defining different age groups would reduce the influence of age on results, however we believe this would require a larger sample and suggest this strategy for future studies.

### Associations between CSF biomarkers and the outcome after shunt surgery

No biomarker could distinguish between improved and non-improved patients. P-tau was the only biomarker associated with outcome; higher levels were associated with less improvement. This association was very weak and should not be used to exclude patients from surgery. Our findings are partly consistent with the results reported by 2015 Nakajima et al., where P-tau levels were significantly associated with the outcome in cognitive symptoms: both pre- and post-surgical levels of P-tau could distinguish between cognitively improved and non-improved patients as measured by change in MMSE scores [37]. Other studies have reported that evidence of AD pathology does not significantly influence outcome negatively [21, 38].

In a review from 2018, Pfanner et al. investigated the role of several biomarkers in the prediction of shunt surgery outcome. It was concluded that Aβ42, Tau, P-tau, NFL and Leucine-rich α-2-glycoprotein (LRG) had the greatest potentials of being significant predictive factors, although none of them held a strong reliability [39]. Hong et al., concluded, in their multicenter prospective study, that a pre-operative low P-tau/Aβ ratio could significantly differentiate shunt-responsive patients from non-responders. The ratio was however not a significant predictor in the regression analysis. Relating this finding to other comorbidities, the authors suggested that the low ratio implied absence of AD pathology and therefore increased improvement rate in these patients [40]. Contrarily, another study concluded that comorbid AD did not significantly correlate with poorer outcome [41].

To rule out possible effects of comorbid AD on associations with outcome, we studied AD-pathology negative patients, defined by A*β*42/A*β*40 ratio, separately but found similar results as in the whole group. Similarly, concentrations of AD-related biomarkers did not differ between improved and unimproved patients. This supports the notion that studied associations mainly reflect iNPH-related disease mechanisms of reversibility.

The responder rate in our patients was 58% which enabled comparison of responders with a large group of non-responders. This response rate is lower than in many contemporary studies. However, we have no reason to believe that this reflects a bias in selection of patients for surgery or the generalizability of results.

### Strengths and limitations

Main strengths of this study are the representative sample iNPH patients and the detailed assessment of clinical symptoms comprising all symptom domains, i.e. gait, balance, cognition and urinary continence. Patients were included at one specialized unit, pre- and post-operatively examined by experienced investigators and operated on at the same center. Diagnosis was based on established diagnostic criteria. Severity of disease was assessed on the iNPH-scale developed specifically for iNPH symptomatology and outcome assessment. This contrasts many earlier studies which have used clinical measures that are crude or not developed for iNPH patients in assessment of symptoms or outcome. Outcome data were available as both ordinal and continuous variables, allowing the statistical analyses to be based on both whether patients were improved or not, and on the extent of the improvement. Analyses of the biomarkers were performed batch-wise by professional analysts blinded to the clinical data.

One limitation of this study is that it includes only patients subjected to shunt surgery based on a diagnosis of iNPH, in some cases supported by a positive outcome of additional predictive testing, which presents a limitation to the ability to generalize the results or to consider using CSF biomarkers for selecting which patients should have shunt surgery. Since we have not looked at patients who were not shunted despite a diagnosis of iNPH, conclusions regarding this group of patients cannot be drawn. However, the use of additional predictive tests in selected cases in this study, probably entails a wider inclusion of treated iNPH patients than if shunt candidates would have been selected based on a positive CSF taptest or infusion test, given the low negative predictive value of these tests [42].

Other limitations include that clinical data of comorbidities were not available and that MRI data as well as other possible confounders were not included. The explorative design was used to reduce the risk of type II errors.

## Conclusions

No CSF biomarker could be used to identify shunt responders or predict outcome, even if there was a weak association between higher P-tau and less pronounced postoperative improvement of limited clinical value. A higher NFL reflecting axonal degeneration was associated with more pronounced iNPH symptoms of gait, balance, cognition and urinary incontinence, whereas higher T-tau and P-tau were associated with poorer cognitive performance. Absence of AD-pathology did not affect these results.

## Data Availability

Data will be made available by the authors upon reasonable request.
